# Ups and downs of addiction harm reduction in Iran: key insights and implications for harm reduction policy and policing

**DOI:** 10.1186/s12954-022-00719-0

**Published:** 2023-01-20

**Authors:** S. Ali Shafiee, AbouAli Vedadhir, Emran Razaghi

**Affiliations:** 1 School of Behavioral Sciences and Mental Health (Tehran Institute of Psychiatry), Iran University of Medical Sciences, Tehran, Iran; 2 Department of Anthropology, Faculty of Social Sciences, University of Tehran, Tehran, Iran; 3 Tehran University of Medical Sciences, Tehran, Iran

**Keywords:** Harm reduction, Policing, Drug policy, Addiction

## Abstract

Drug use is a critical behavioral disorder or a delinquency behavior (in the judiciary system's words) that comes with a burden at multiple levels: individual, community, public, and global. These social structures apply different interventions to reduce this burden in their field. Given the society as a whole, these structures must be harmonious and synergistic to optimize these endeavors in terms of cost–benefit. In practice, however, reducing the burden of addiction is followed by conflicting approaches by different organizations, in terms such as “eradicating drugs,” “eliminating drug users,” “obliterating addiction,” and ultimately, drug use harm reduction. In the harm reduction philosophy, drug use is recognized as an inescapable fact in human societies, and tries to control its personal and public consequences in different dimensions (health, economic, and social). Therefore, this approach includes broad measures such as: changing the pattern of consumption (from high-risk substances to less dangerous substances) through modification of the laws and law enforcement measures, distributing disposable syringes to prevent HIV transmission, providing basic life needs such as shelter for street-based drug users to reduce the social consequences like homelessness, prescribing substitute agonists to reduce the committing crime to obtain the needed drugs, and even the drug court program, which prevents the exacerbation of complications in a person with a chronic and relapsing disorder, due to the imposition of inappropriate sentences (like incarceration in unacceptable conditions). It is contrary to the approaches that aim to reduce the drug supply rate and drug use incidence and prevalence to zero. As a result of the conflict of interests, goals discrepancies, and differences in organizational culture, these approaches may contrast with each other. We see this in the harm reduction between the health system and law enforcement. Different factors affect the harmony or conflict between these two structures. This article addresses the impact of ideology, social conditions, and bureaucratic administration on the relationship between the health system and the police in drug use harm reduction in Iran.

## Background and context

In the last half-century, Iran has experienced five different areas of dealing with the addiction problem. About one decade before the 1979 revolution in Iran, when heroin use, like in many other parts of the world, had become a health and social problem [1], law enforcement focused on implementing rules enacted based on the international drug control conventions that highlighted supply reduction [2].

### *One*

Decades ago (in the 1930s), the regime, like the hijab and other traditional clothing, recognized narcotic use as an obstacle to authoritarian development. The regime forced drug users to cease, made women remove their hijab (unveiling) and men wear clothes according to Western fashion [3]. In line with the latest scientific findings, addiction was gradually accepted as a disease that needed to be treated by specialists. Although those who had not quit using drugs were subject to social penalties, the law had not criminalized drug use, and, as a medically harmful condition, the authority and responsibility for managing the "problem of addicts" had been delegated to the Ministry of Health and Medical Education (MOHME). However, this policy did not provide adequate addiction treatment coverage in Iran [2].

With no harm reduction approach but to decrease smuggled drugs, the government in pre-revolutionary Iran started to locate opium rations for the elderly dependent users in 1956 [2]. This measure did not solve the problems induced by the rising trend of heroin use among the youth and caused the leak of government opium to the black market for the consumption of other people. It was not law enforcement, but health professionals who criticized this policy, perhaps because this approach's legal and security consequences were lesser than illegal drug trafficking by violent criminals, and the police prefered to focus on drug supply reduction. As a result, there was no significant challenge between the health system and law enforcement. However, the imbalance of supply and demand reduction policies and ignoring the dynamics of these two areas was one of the critical reasons for the failure of efforts for the first maintenance program in Iran [4].

### *Two*

Almost all anti-monarchist revolutionaries, regardless of their ideology and slogans, raised this policy as a sign of the regime's deliberate attempt to narcotize the masses and normalize Western-style liberalism. They saw the prevalence of drug use as one of the imperialist tricks to weaken independent societies and defeat the revolution's ideals. Hence, it was no surprise that shortly after the 1979 Islamic revolution, the new Islamic government cut opium quotas, criminalized drug use, and mandated addicts to quit drug use within six months [5].

Afterward, the Revolutionary Courts became responsible for dealing with drug-related crimes [6]. Prosecuting perpetrators of imprisonment, torture, and murder of revolutionaries in the former regime, as well as prosecuting acts such as assassination, sabotage, espionage, and economic conspiracy against the Islamic Republic regime, are these courts' duties. Also, they deal with drug-related crimes, and within a week, they must issue a sentence that is not appealable [7].

Central law enforcement of these courts was the Islamic Revolutionary Committees, one of the first institutions established after the revolution, to obtain security and discipline in the country to protect the achievements of the Islamic Revolution of Iran [8]. In addition to intelligence and security missions and fighting against the counter-revolutionaries, the committees were responsible for maintaining the moral security of society against depravities, including drinking, and non-observances of the Hijab law, illegitimate relations between men and women, supply and the consumption of alcohol and drugs [9]. Nearly twelve years later, these committees merged with two different institutions in terms of organizational structure and objectives, namely the civilian police and the gendarmerie, to form the police force of today, with committees playing a fundamental role, especially in its command ranks.

The main idea at that time was to quickly and decisively eliminate all the problems and deprivations attributed to the former regime by using the revolutionary will in the direction of the revolution's ideals. In an endeavor to fight against drugs, the revolutionary courts and committees carried out this determination to “eradicate drugs” and “eliminate drug users” from society through two principal measures: The imposition of severe punishments, such as execution and long-term imprisonment for both drug dealing and trafficking; and, collecting drug users and keeping them in compulsory camps to the forced cessation of drug use [5]. Despite the efforts of the health care system to develop addiction treatment in the country, the nature of the addiction disease did not allow most consumers to permanently quit drug use within the six months prescribed by the law. However, due to ideological beliefs, law enforcement had no restrictions on performing “the sacred duty of cleansing society of the contamination of addicts.” Thus, they started mass arresting drug users and imprisoning them before the end of the six-month legal deadline for quitting addiction [10]. It is the route of deep conflict between the health and judicial systems.

The concept of addiction as a chronic, recurrent, and treatable disease; and the notion of substance use as a perverted, sinful, criminal, and counter-revolutionary conduct, which must be repented of or punished, are in contrast. Naturally, forming a harm reduction approach to substance use in such an environment was impossible. The law, which originated from a jurisprudential-revolutionary ideology, obliged drug users to quit drug use and law enforcement to purge society of addicts; then, dissidents could not raise other diverse approaches, such as harm reduction.

### *Three*

This situation continued until the pragmatic government came to power after the devastating eight-year Iran–Iraq war (1980–1988). The effects of the war and the consequences of socioeconomic transformations changed the patterns of drug use, drug users, and the harms of addiction in Iran. The harm could not be controlled by gathering arrested addicts to force them to quit drug use in compulsory camps and forsaken islands of the Persian Gulf. Then, it allowed the government to raise reconsideration of counter-narcotics policies at the highest levels of authority, the Expediency Discernment Council.

The new 1997 amendment still criminalized drug use and forced addicts to quit to avoid punishment. However, two decades after the revolution, drug users with no deadline were allowed to take addiction treatment and rehabilitation at medical centers licensed by the MOHME [11]. Nevertheless, treatment in government facilities was inadequate and limited to detoxification. In this period, harm reduction still had no place inlaws or scientific societies, and even some experts were skeptical about it.

### *Four*

What changed the scene was the prevalence of HIV infection among people who inject drugs (PWID), particularly in prisons [12]. The danger of HIV was previously ignored by the Islamic Republic of Iran and attributed to unrestrained sexual relationships in Western countries. However, it finally emerged through the gates of addiction.

Health care system technocrats, first in the field of infectious diseases, then mental health and addiction, turned this threat into an opportunity to build the foundations of addiction harm reduction programs in Iran. Their advocacy efforts were fantastic, and they could get the judiciary's support at the highest level. The health care system developed NSP, VCT, then MMT programs in prisons and high-risk areas [13, 14]. Despite legal requirements, judicial authorities prohibited the imprisonment of individuals solely on charges of addiction [15]. Shortly after, the words "harm reduction" entered the Iranian legal literature through the Anti-Narcotics Law adopted in 2010 [16].

Although in the beginning, even some judicial authorities persecuted harm reduction activists and even the IMOH officials on charges such as abetment in the crime of drug use, with the emergence of harm reduction benefits, their views on these programs changed.^1^ The MMT program has reduced everyday struggles over drug possession in prisons as much as it is impossible to imagine a prison without the MMT program. Also, social support and harm reduction programs have decreased drug-seeking conduct and illegal behaviors to get drugs. Furthermore, thousands of people covered by agonist drugs, especially MMT, have been removed from the criminal population and returned to ordinary life for not committing drug offenses. Finally, the decline in the incidence of HIV/AIDS among injecting drug users in Iran presents the world as a successful example of the health and judicial systems' participation in harm reduction programs [17].

All of these took place in the context of socio-political changes in which reformism, tolerance, and civil society were the main keywords. That is everything necessary for the harm reduction movement in Iran. Under these circumstances, not only did the NGOs take a humane approach to modify strict policies in dealing with drug users, but also the police, who were trying to show a changed face in line with the new social atmosphere associated with this movement [18].

The police carried out the orders of the judicial authorities regarding harm reduction activities and dealing with their clients. However, the Iran Drug Control Headquarters (DCHQ), the highest executive authority in this field, played an essential role in bringing together the government bodies with different opinions and interests, including the police and health system [19]. At the time, balancing the measures of reduction of drug supply and demand was the central approach in global drug policy [20]. Then, the DCHQ sought to lead the activities of various organizations in these areas in a direction that met the goals of the general policies of the system in drug control. Although the general drug policy still criminalizes drug use, it excludes the use of psychotropic medications (such as opioid agonists) for drug treatment and harm reduction. In particular, it considers preventing the change of consumption patterns from low-risk to high-risk substances as one of the main goals [21].

### *Five*

The honeymoon was short for Iran's drug use harm reduction. With the entry of stimulants (in the mid-2000s) into the Iran drug market, a new pattern of consumers appeared with distinct demographic and socioeconomic characteristics from usual opioid users [22]. This pattern of use caused unprecedented problems of drug addiction. The media exaggerated the noisy consequences of stimulant use [23], including aggressive behaviors, domestic violations, violent crimes, and its rapidly destructive effects, and panicked the community; the harm reduction managers were also surprised by the new situation [24]. Before these problems were associated with the intrinsic properties of stimulants, they were the result of marginal factors such as the impurity of drugs available in the market and consumers' unfamiliarity with new substances to control consumption to reduce its side effects. Over time, users of stimulants have become aware of how to manage the use of these substances, and harm reduction infrastructures have significantly impacted creating this awareness; stimulants are no longer an exciting subject for the media!

Reducing the risk of HIV transmission through injecting drugs has been the central goal of addiction harm reduction programs in Iran for two decades, and the health administration has focused on achieving this goal. Therefore, the "addiction harm reduction approach" in Iran has been reduced to interventions to "reduce the risk of HIV transmission through injecting drug use." For many reasons, including the training provided through harm reduction programs, the prevalence and incidence of injecting drug use are much lower today than in the past [17]. However, in the harm reduction package, the necessary interventions have not been prepared for other consequences of drug use, especially at the social level. As the social effects of stimulant use were greater than those of narcotics, the weakness of harm reduction programs in dealing with these substances became more apparent.

The burden of these social consequences on the judiciary and law enforcement increased even more. They directly faced considerable people committing crimes under the psychotic effects of drug use that the health system had no plan to manage. This unexpected situation made doubt the potential of harm reduction programs. Gradually, the police, who were at the forefront of dealing with new challenges, reduced their vital involvement with the health system for harm reduction programs.

These changes coincided with the end of the work of the reformist government in 2005, which advocated harm reduction, and the emergence of a government with reactionary ideas and a revolutionary manner that sought to solve social, economic, and other problems by instant plans. The changes were not only at the political level but also altered the social and economic situation of the country, which directly or indirectly affected the quantity and quality of government health and support services and the participation of non-governmental sectors in providing them [19]. Similarly, policies on dealing with drug users, especially street addicts, the target group of harm reduction interventions, changed. According to the Anti-Narcotics Law, they are taken to compulsory camps and even to jails by police on charges of exposing manifestations of drug use and addiction in society [16].

## Discussion

The dominant way of HIV transmission has now changed from injecting drug use to unprotected sex. However, Iran's AIDS-based harm reduction program ignores other public health harms such as declining mental health, accidents, poisoning, and overdose  deaths. Instead, other public problems, such as insecurity and the sense of public insecurity caused by the presence of street addicts, have become the main harms of addiction in the eyes of society. The problems which fall within the scope of police missions and their situation are known as the indicator of police performance evaluation. The police institution cannot fundamentally control drug users' petty crimes and offenses rooted in the deprivation of proper support, treatment, and harm reduction services for drug users. Hence, to maintain its credibility and reduce the burden of expectations, it inevitably “eliminates addicts” from the public space. As a result, we face a vicious cycle. Society reproduces street addicts constantly, and the forced cessation of drug use merely deepens the deprivation of in-camp incarcerated addicts from accessing the services they need. The outcome is an increase in the burden of addiction due to its various individual and public harms, including drug-related crimes. This means that the more police mission programs conflict with the harm reduction services, the more health problems and the more burden for the police.

What can break this vicious cycle is changing society's attitudes at all levels toward the concept of security and sustainable ways of providing it. Suppose the security of a group in society, even if it is a minority, is distorted as a fundamental right. In that case, other social groups' rights (including security) will also be threatened. Society must realize that sustainable security is only achieved if all social groups, including those who may seem different from the majority or called "the others," are safe. Hence, when a citizen is confronted with a homeless citizen who uses drugs, instead of the police, s/he will call the social security service or a familiar supporting NGO. This sociocultural awareness is achieved through formal education and the media.

When social demonstrations of addiction become the pretext of political competition, pressure mounts on the government, and a conservative government prefers to expel street addicts from public view by police forces to escape the reproaches of political opponents. These pressures can come directly to the police from political authorities or the media, and highly interpretable law allows law enforcement to exercise power over this voiceless population, people who need harm reduction services. This is what we saw between 2013 and 2017 when the centrist government was pitted against conservative political rivals in parliament and the judiciary.

In recent decades, the over-criminalization of citizens' behaviors in Iran's criminal law [25], consuming massive human resources and facilities, has involved the police in the various parts of the daily life of Iranians, from the hijab to the use of satellite receivers. Accordingly, the lack of required resources prevents police and judicial from specialized proceedings of drug-related crimes (such as drug court programs); despite the legal requirements for effective tailored measures, the system treats all addicted people as a homogenous mass.

Spending vast resources on the forced quit addiction of these people in the compulsory camps has had no effect other than temporarily preventing their delinquency during incarceration. This inefficient approach to a multidimensional problem has constantly increased the volume of this mass as a part of the criminal population and raised its social, economic, security, and health consequences at the individual and public levels.

Iran is on the transit route of international opiate trafficking due to its proximity to Afghanistan, the world's largest producer of opium; it suffers a lot of human and financial losses to fight this global problem. Even in the most challenging international relations, other countries and international bodies have appreciated Iran's efforts in this struggle (UNODC Drug Reports). The criterion for countries' commitment to international conventions to reduce the global supply of narcotics is the volume of illicit drugs they seize. However, the benchmark for police success in combating drug supply is reduced access to drugs (especially high-risk drugs) nationwide [26]. At the same time, there is not enough scientific evidence to show the impact of supplier arrest and seizure on a range of drug-related outcomes [27].

Iran may not be the only example, where its internal affairs and law enforcement officials see the presence of street addicts in public as a source of public doubt about the government's commitment to the fight against drugs. They consider the public gathering of drug users, even to receive harm reduction services in DICs, as a sign of the government's failure to “addiction eradication,” as an ideological goal that they more try to achieve, the more unattainable it becomes. So now that "addiction eradication" in general and drug dependence, in particular, is not possible, the government focuses on physically “eliminating drug users” from the public space to regain self-confidence and prestige. In the meantime, street addicts are receiving more attention due to their symbolic aspect as a sign of government disability. This issue has been so crucial to the police (and IRGC) that they directly keep some arrested addicted people in detention centers and former barracks under their supervision. The rest are held in prisons, rented private camps, and government compulsory residential centers.

## Conclusion

The above overview reveals that the three main components in Iran have affected the relationship between the law enforcement forces and the addiction phenomenon in general and harm reduction programs in particular.

*1. Ideology:* Even before the 1979 Islamic revolution, the regimes’ ideological orientations influenced the addiction issue (Recall the simultaneity of the policy of obligatory unveiling and dealing with drug use with the police force). The authoritarian development of the first Pahlavi, Reza Shah Pahlavi (1925–1941), required the violent confrontation of the police with addiction as an obstacle to the progress of the country and addicts as the symbols of backwardness. The medicalization of addiction in the second Pahlavi, Mohammad Reza shah period (1941–1979) also originated from Western-style modernization, which sought to subjugate a culture-driven behavior with experimental tools. In a situation where the pattern of substance use was changing to heroin, allocating the opium quota for some addicted people could not, as a health intervention, have the harm reduction effect. Since it was thought that this action would reduce the opium smuggling from neighboring countries into Iran (and the outflow of the country's funds to buy it), the judicial system and the police supported this plan [2].

The ideological approach to drug use, drug users, and addiction became more pronounced after the 1979 revolution [28] to the extent that the law enforcement against this phenomenon was the institution (The Revolutionary Committees) whose task was to detect and thwart all kinds of conspiracies against the revolution.

The integration of law enforcement agencies into the police during the postwar reconstruction period (1991) was a pragmatic move that could have moderated the dealing with drug users. However, considering the aggravation of drug-induced harms as an inevitable but negligible complication of the development, the war on drugs and drug users prevailed over other approaches in that period. In wars, there is no place for peaceful measures such as harm reduction, and law enforcement is the guarantor of this irreconcilability.

Ultimately, the opinions of the reformist movement were strengthened by the victory in the 1997 presidential election and made possible the harm reduction approach to addiction in Iran. The tolerance of the opposition had grown so strong in the community that it forced the police to show the necessary flexibility to carry out these interventions. Also, paying attention to civil society enabled the growth of the relevant NGOs active in harm reduction as the leading providers of these services under tolerance and even protection of the police. However, a decade and a half later, the weak theoretical underpinnings of harm reduction in Iran defeated it in the face of a conservative discourse that pursued the war on drugs and drug users by proxy of police in an institutional rather than ideological manner.

This experience shows that harm reduction is not just a negative concept to confronting the criminalization of addiction but philosophical theorizing of the addiction phenomenon with an affirmative approach. Then, continuing evidence-based dialogue about various aspects of the phenomenon and scientific critics of harm reduction is vital [29].

*2. Society:* If the policy of suppressing substance users is related to the pre-modern age and the treatment and rehabilitation approach to drug use disorders belongs to the modern era, then addiction harm reduction is a postmodern concept. Harm reduction is not an imported fashion that changes the public taste for a while and then gives way to another. Still, it is a product of society's insight and a sign of its tact in dealing with complex social issues. Harm reduction providers and law enforcement are both parts of this society, and what happens in the field between them is a result of the social condition of the day.

Even in less democratic societies, whether the public demand is for street-based addicts to be collected by the police or supported by social institutions will be determinative in the government's drug policy. In democracies, the community's sense of responsibility and participation in the phenomenon of addiction reinforces the necessary empathy in law enforcement forces to accompany harm reduction interventions. However, the instability of a society in transition can sometimes be so distressing that the community will force to abandon empathy with others as a psycho-social need to obtain security as a basic need. Such a society cannot be sensitive to using unbridled force against the stigmatized harm reduction target group.

The way out of this vicious cycle is broad and deep public awareness so that instead of demanding the police sweep the problem under the carpet, society responsibly requires the health, treatment, and social support authorities to optimally and humanely manage drug use harms. Also, it is necessary to train law enforcement continuously about the goals and techniques of addiction harm reduction and its benefits for society, including providing stable security.

*3. Administration:* The medicalization of addiction before and after the 1979 revolution and the harm reduction interventions in Iran in the late 1990s and early 2000s were the initiatives of talented technocrats in the executive body of the government in the areas of social support, health, and drug control. Unlike their conservative predecessors and descendants, they approached the addiction problem from a new perspective. The harm reduction pioneers indeed turned the threat of HIV/AIDS into an opportunity to develop harm reduction programs. To this end, they acquired extensive advocacy from various parts of the system, especially the judiciary as a higher law enforcement body. They then obtained the collaboration of key persons in the police to implement the harm reduction program.

Police commanders had the same desire for their organization's structural and functional development and to improve its image in society. Their previous and next counterparts' symbolic goal was to save even one addict in the sense of eternal purity from drug use at any cost. However, by cost–benefit calculation, they preferred the long-term benefits of the harm reduction approach in reducing the social burden of addiction, including drug-related crimes, to the temporary situation control of the crimes through the “social elimination of drug users.”

After the leading creative technocrats in addiction harm reduction gave way to conservative managers, the advocacy necessary for the continued support of the judicial and law enforcement system for these programs was severely weakened, and the initiative was taken away from the health and social support system. This history reminds us of the importance of tireless advocacy for Harm Redaction.

Although police in Iran are part of the armed forces, due to the nature of its missions, it has had a close relationship with the established governments. Thus, the dominant discourse of the incumbent government and its bureaucrats influences them. However, the pace of change in government institutions is faster than in law enforcers. Thus, when a conservative government, which does not attach much importance to avant-garde approaches such as harm reduction, replaces the transformational government, new authorities allocate less financial, human, political, and administrative resources to previous progressive programs.

Governments change every four or eight years with the slogan of transformation in matters of public concern, while the missions of the judiciary and the police change less over time. Therefore, we can expect these institutions to be the starters of the fundamental changes needed for harm reduction programs or their foremost advocates; if harm reduction is so politically and socially institutionalized, ignoring it would be costly to shift to a new equilibrium point for all actors.

This shows the importance of organizations outside the government and political parties to advocate harm reduction policy and demand it from the judicial and law enforcement system. Suppose the international institutions had encouraged Iran's police to participate in national harm reduction programs as much as they praise it for drug seizing. In that case, Iran's police could be a role model for other similar countries. One of them is Afghanistan, which is related to Iran not only in terms of drug consumption patterns but also in terms of language and culture. The promotion of harm reduction programs and, generally, demand reduction policies in Iran can directly and indirectly affect Afghanistan and lead to a significant reduction in drug supply from this largest narcotic proucer in the world.

Harm reduction is not a tactic or even a strategy in the face of the problems caused by drug use but a conceptual approach to the complex phenomenon of addiction that direct methods cannot control the consequences of it. Because the burden of these consequences is multidimensional (biological, psychological, and social) and multilevel (individual and public), the typical policies that address only one dimension or one level of harm, such as public health or social security, have limited success. Therefore, to reduce the burden of addiction, we need a package of comprehensive, continuous, and integrated interventions that include all the harms of substance abuse and addiction. The variety of harms makes multi/inter-sectoral cooperation inevitable. This cooperation requires a mutual understanding of sectors with different characteristics from the considerations of others.

It is natural for diverse sectors such as the health system, the judiciary, and the police to have different priorities and concerns in dealing with addiction, to have conflicts of interest, and compete for more resources to address the consequences of drug use in their field. However, it would be disappointing if these conflicts and competitions increase the burden of addiction. Shared ideals, an informed society, and an intelligent administration can turn the threat posed by these differences into an opportunity to optimize harm reduction interventions to minimize the burden of addiction.

## Data Availability

Data sharing is not applicable to this article as no datasets were generated or analyzed during the current study.

## Footnotes

1 On January 17, 2005, the head of the judiciary, through a circular, warned the judicial authorities not to charge people active in harm reduction programs with the crime of assistance in drug use due to the distribution of sterile syringes and needles or the administration of methadone.
